# Masked Channel Modeling Enables Vision Transformers to Learn Better Semantics

**DOI:** 10.3390/e27080794

**Published:** 2025-07-25

**Authors:** Jiayi Chen, Yanbiao Ma, Wei Dai, Zhihao Li

**Affiliations:** 1 School of Telecommunications Engineering, Xidian University, Xi’an 710071, China; 22012100031@stu.xidian.edu.cn (J.C.); 22012100039@stu.xidian.edu.cn (W.D.); 2 School of Artificial Intelligence, Xidian University, Xi’an 710071, China; anna383960@163.com

**Keywords:** Masked Channel Modeling, CLIP target, semantic continuity

## Abstract

Leveraging the ability of Vision Transformers (ViTs) to model contextual information across spatial patches, Masked Image Modeling (MIM) has emerged as a successful pre-training paradigm for visual representation learning by masking parts of the input and reconstructing the original image. However, this characteristic of ViTs has led many existing MIM methods to focus primarily on spatial patch reconstruction, overlooking the importance of semantic continuity in the channel dimension. Therefore, we propose a novel Masked Channel Modeling (MCM) pre-training paradigm, which reconstructs masked channel features using the contextual information from unmasked channels, thereby enhancing the model’s understanding of images from the perspective of channel semantic continuity. Considering that traditional RGB reconstruction targets lack sufficient semantic attributes in the channel dimension, MCM introduces advanced features extracted by the CLIP image encoder as reconstruction targets. This guides the model to better capture semantic continuity across feature channels. Extensive experiments on downstream tasks, including image classification, object detection, and semantic segmentation, demonstrate the effectiveness and superiority of MCM. Our code will be available later.

## 1. Introduction

Since the success of Masked Language Modeling [1] in the natural language processing field, Masked Image Modeling (MIM) [2,3] has gradually become a mainstream pre-training paradigm for visual representation learning in the computer vision field. By masking parts of the input and reconstructing the original image, MIM pre-trained Vision Transformers (ViTs) [4,5] are able to learn rich visual representations, significantly improving performance in downstream classification, detection, and segmentation tasks. Thanks to their long-range modeling capabilities, ViTs can capture global contextual information within images and effectively model relationships between different spatial patches, thereby further advancing the development of MIM in visual representation learning.

**Figure 1:**
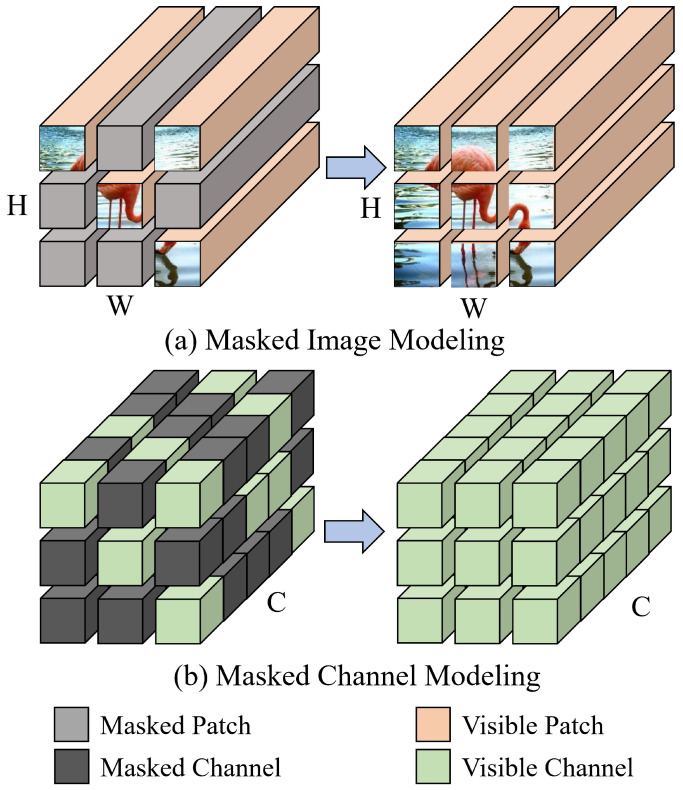
(**a**) The image size is denoted as (H, W, C), referring to height, width, and channels.Most existing MIM methods emphasize spatial continuity by focusing solely on pixel reconstruction within patches. (**b**) The proposed MCM paradigm leverages the semantic information of unmasked channels to reconstruct the features of masked channels, compelling the model to account for semantic continuity along the channel dimension.

For general Vision Transformers, the features extracted from a specific spatial patch of an image should reflect a certain level of spatial continuity [4,6]. Simultaneously, channels within that patch that exhibit semantic continuity should be able to map to specific objects or semantic concepts [7]. This ability allows ViTs to accurately capture the semantic structure of an image, thus promoting a deeper understanding of image semantics and facilitating cross-modal information alignment. However, as illustrated in Figure 1a, most existing MIM methods [2,3,8] focus primarily on spatial patch-level reconstruction, attempting to learn both local and global visual representations by emphasizing spatial continuity. They overlook the importance of semantic continuity in the channel dimension, specifically the transmission relationships of semantic information between channels within the same patch or across different patches. To address this limitation, as depicted in Figure 1b, this paper proposes a new Masked Channel Modeling (MCM) paradigm, which leverages the contextual semantic information from unmasked channels to reconstruct the features of masked channels, strengthening the model’s understanding of channel semantic continuity and enriching its representational capability.

Following the classic asymmetric encoder–decoder architecture of MAE [3], MCM first randomly masks a large proportion of the channels (e.g. 75%) in each patch and replaces the masked channels with the shared and learnable encode token. These embeddings are fed into a ViT [5] encoder, followed by the decoder to complete channel reconstruction. Unlike traditional methods that use pixel-based targets, this paper introduces advanced features extracted by the CLIP image encoder [9] as the reconstruction target [10,11]. The CLIP advanced features are closely associated with semantic information in each channel, such as object categories, attributes, and contextual relationships. This design effectively overcomes the limitations of traditional pixel-based targets, which lack sufficient channel semantic attributes, enabling the model to learn deeper semantic relationships.

By shifting the focus of modeling to the channel dimension, MCM is able to better capture the semantic continuity between feature channels and benefit from more fine-grained semantic information, such as the diversity of objects, contextual relationships in the background, and the decoupling of different semantic features [12,13]. Extensive experiments have demonstrated that MCM shows significant advantages in downstream image classification, object detection, and semantic segmentation, validating the crucial role of channel semantic continuity in enhancing the model’s representational capabilities.

Masked Image Modeling (MIM), such as MAE, primarily captures spatial relationships by reconstructing masked image patches, but may overlook the deeper semantic continuity among channels, often confusing visually similar yet semantically distinct regions or objects. To address this limitation, we propose Masked Channel Modeling (MCM), which explicitly targets semantic continuity across channel dimensions, compelling the model to infer missing semantic features from the remaining visible channels.

## 2. Related Work

### 2.1. Masked Image Modeling (MIM)

MIM techniques, such as MAE [3] and BEiT [2], focus primarily on spatial continuity. Further variants, like MaskFeat and MVP, introduced semantic-rich reconstruction targets, improving high-level representation learning.

### 2.2. Masked Channel Modeling (MCM)

Liu et al. [14] recently proposed an MCM method specifically utilizing pre-trained CLIP visual backbones, directly reconstructing masked CLIP feature maps. Chau Pham et al. [15] introduced ChA-MAEViT, tailored explicitly for multi-channel image data, promoting cross-channel feature dependency learning.

Our MCM differs distinctly: rather than fine-tuning a pre-trained CLIP model or focusing on multi-channel spectral data, we employ a general MAE-style ViT encoder–decoder architecture trained from scratch, using CLIP features strictly as semantic reconstruction targets. This ensures broader applicability and more effective general semantic feature learning.

## 3. Masked Channel Modeling

**Figure 2:**
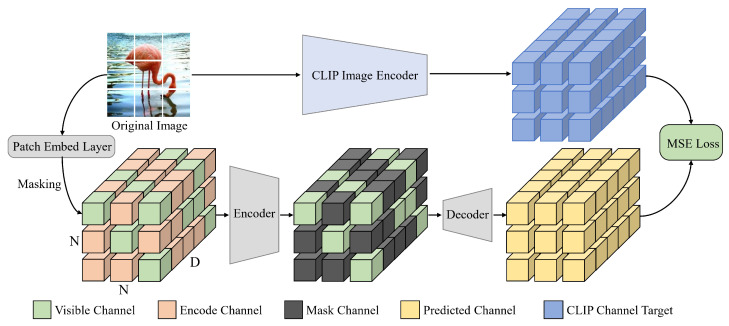
The pipeline of MCM. Given the N×N patch embeddings of dimension *D* obtained through patchified, we randomly mask the channels of each embedding with the same ratio. The masked channels are replaced with a learnable and shared encode channel to ensure alignment between the number of input embedding channels and the requirements of the Vision Transformer blocks. Before decoding, these encode channels are replaced with another set of independent mask channels. Finally, MCM uses the features extracted by the CLIP image encoder from the input image as reconstruction targets to guide the model in learning better high-level semantics.

The MCM pipeline is illustrated in Figure 2. We follow the classic MAE [3] encoder–decoder asymmetric architecture, using a standard Vision Transformer (ViT) [5] as the encoder and two layers of 768-dimensional ViT blocks as the decoder. Through a random masking strategy, a portion of the channels in each patch embedding is truncated. MCM aims to leverage contextual information from unmasked channels of the same patch or neighboring patch embeddings to predict the masked channels, thus forcing the model to focus on semantic continuity in channels. In addition, MCM uses the advanced features extracted by the CLIP image encoder from the input image as reconstruction targets, ensuring that the model learns high-level semantics and demonstrates stronger discriminative ability in downstream tasks.

**Encoding.** The input image $I\in R^{H\times W\times C}$ is first patchified into a series of non-overlapping patches of size p×p, which are then mapped into patch embeddings $x\in R^{N\times D}$ through a linear layer, where $N=HW/p^{2}$ is the number of patches and *D* is the number of channels for each patch embedding. We randomly mask a proportion γ (e.g., 75%) of the channels $D_{m}$ of each patch embedding, ensuring that the remaining unmasked portion of each embedding (e.g., 25%) retains the same number of visible channels $D_{v}$. Unlike traditional MIM, which discards the masked patch $x_{m}\in R^{N_{m}\times D}$ and only inputs the visible portion $x_{v}\in R^{N_{v}\times D}$ into the encoder, MCM replaces the masked channels with a shared and learnable encode channel $e\in R^{N\times D_{m}}$. This ensures that the total number of input embeddings $\left\{x,e\right\}\in R^{N\times (D_{v}+D_{m})}$ aligns with the required number of channels *D* for computing multi-head attention in each standard ViT block of the encoder, where $D=D_{v}+D_{m}$.

**Decoding.** Before entering the decoder, if the encode channel $f_{e}\in R^{N\times D_{m}}$ of the embeddings $\left\{f,f_{e}\right\}\in R^{N\times (D_{v}+D_{m})}$ extracted by the encoder is retained, the model may memorize the encoded values, reducing its reliance on contextual information during reconstruction. This information leakage significantly decreases the model’s learning efficiency and sensitivity to the masked information. To address this, we replace $f_{e}$ with another independent set of shared and learnable mask channels $m\in R^{N\times D_{m}}$. Finally, the modified embeddings $\left\{f,m\right\}\in R^{N\times (D_{v}+D_{m})}$ are fed into the decoder, which outputs $z\in R^{N\times D}$ for reconstruction.

**CLIP target.** Considering that raw pixels lack explicit deep semantic attributes and cannot effectively guide the model to learn representations with semantic continuity, MCM adopts high-level semantic features from CLIP [9], which are highly discriminative and exhibit strong cross-modal consistency, as reconstruction targets. Specifically, the input image $I\in R^{H\times W\times C}$ is first fed into the CLIP transformer-based visual encoder G(·) to extract advanced features $g_{I}\in R^{N\times D_{c}}$. These features are then passed through a simple linear layer to map $g_{I}$ to *D*-dimensional space, resulting in ${\hat{g}}_{I}\in R^{N\times D}$, ensuring that the CLIP target aligns dimensionally with the decoder’s predictions. Notably, MCM reconstructs the semantics of all channels, not just the masked channels, using the CLIP features as the target. This is achieved by calculating the Mean Squared Error (MSE) loss as follows:

(1) $$L_{MSE}\left(z,{\hat{g}}_{I}\right)={∥z-{\hat{g}}_{I}∥}_{2}^{2},\;$$

When reconstructing masked channels, the model generates approximate target features by leveraging the contextual information from unmasked channels. The high-level semantic features from CLIP ensure that the reconstructed channels exhibit semantic continuity with their neighboring channels, thereby guiding the model to learn more discriminative representations. Compared with feature mimicking approaches such as ref. [16], our method differs fundamentally in the training objective. While ref. [16] imposes supervision on visible tokens using external pre-trained features, our method formulates masked channel modeling as a self-contained reconstruction task across feature channels, encouraging semantic continuity without direct mimicking losses. This shifts the learning dynamics and facilitates different types of representation structures.

## 4. Experiment

### 4.1. Experimental Setups

Similar to the evaluation protocol of [2,3], our MCM model is fine-tuned on classification, detection, and segmentation tasks. Unless otherwise stated, all experiments are implemented on the PyTorch 2.1.0 platform and conducted on eight 24 GB RTX 3090 GPUs (NVIDIA Corporation, Santa Clara, CA, USA). For detailed experimental settings, please refer to the Appendix A.

**ImageNet-1k [17].** For pre-training, the training set of ImageNet-1k is used for visual representation learning. Each image is resized to 224×224 pixels. MCM employs a plain ViT-B/16 [5] as the encoder and a decoder consisting of two layers of 768-dimensional ViT blocks. The random masking ratio γ is set to 75%. Except for the batchsize of 1024, other pre-training configurations follow [3].

For the classification task, we report the Top-1 accuracy on the ImageNet-1k validation set with a fine-tuning protocol following previous practices [2,3]. Fine-tuning is performed with a batchsize of 1024 for 100 epochs.

**COCO [18].** Experiments for detection and segmentation are conducted on COCO. We take Mask R-CNN [19] with FPN [20] as the detector with 1024 × 1024 resolution, following common practices [3,16]. We end-to-end fine-tune for 1× schedule (12 epochs) with the batchsize of 8. The other hyper-parameters follow the default settings of the detector. We report box AP ($AP^{b}$) for object detection and mask AP ($AP^{m}$) for instance segmentation. The above experiments are implemented by the detectron2 [21] and the ViTDet [22] codebases.

**ADE20k [23].** Semantic segmentation experiments are conducted using the UperNet [24] framework for end-to-end supervised fine-tuning on ADE20K, with 512 × 512 resolution. The UperNet is trained for 160k iterations with a batch size of 16, while other hyper-parameters remain at the default settings. The mean Intersection over Union (mIoU) on the ADE20K validation set is reported. These experiments are implemented using the mmsegmentation [25] library.

### 4.2. Main Results

To demonstrate the competitiveness of MCM, we selected 14 different pre-training methods for comparison, including classic contrastive learning methods MoCo v3 [26] and DINO [27], classic MIM methods BEiT [2], MAE [3], and SimMIM [28], HOG feature-based reconstruction methods MaskFeat [8] and LocalMIM [29], attention- and frequency-based reconstruction methods SemMAE [30] and PixMIM [31], spatial patch-level CLIP feature reconstruction methods MVP [32], MILAN [33], and MR-MAE [16], as well as methods combining contrastive learning and MIM, such as iBOT [34] and BootMAE [35].

**ImageNet-1k classification.** Table 1 shows that MCM, pre-trained for 300 epochs, outperforms all methods except MILAN and MR-MAE. MCM pre-trained for 1600 epochs achieves the best results, even surpassing MR-MAE with ConvViT [36] as the backbone and MILAN which incorporates attention sampling [37]. These methods have more complex architectures, whereas MCM, with its simpler structure, learns effective high-level semantics, helping the model make better predictions, fully demonstrating the effectiveness of MCM.

**COCO detection and segmentation.** In Table 1, MCM pre-trained for 300 epochs surpasses MoCo v3 and MAE methods with similar pre-training epochs. As training progresses, MCM with 1600 epochs achieves the best performance, with object detection results matching those of MR-MAE, while instance segmentation results improve by 0.2 $AP^{m}$. More training epochs fully unlock MCM’s potential, helping the model capture contextual information between channels and enhancing a better understanding of the relationships between different objects and the overall scene semantics.

**ADE20k semantic segmentation**. In Table 1, although MCM is only superior to MAE after 300 epochs of pre-training, with more epochs, the version pre-trained for 1600 epochs achieves the best semantic segmentation results, improving by 0.9 mIoU compared to the second-best MR-MAE. We attribute this significant improvement to the MCM paradigm, which enhances the model’s ability to perform collaborative modeling across all channels, enabling a better understanding of both the global and local semantics of the image.

### 4.3. Ablation Study

**Masking ratio γ.** The results for masking different ratios of patch embedding channels are shown in Table 2. Since the COCO performance at γ=80% is significantly lower than that at γ=75%, we set γ=75% as the default ratio.

**Decoder setups.** Table 3 presents the results with different numbers of ViT blocks in our decoder. The configuration with two blocks achieves the best performance. Having too many blocks disrupts the model from reconstructing CLIP high-level semantics, leading to overfitting on local details, which negatively impacts fine-tuning for downstream tasks.

**Reconstruction target.** Table 4 provides the results of MCM reconstructing different targets in various ways. When reconstructing pixel-level spatial patches, MCM degenerates into a simple MAE [3]. Reconstructing pixel-level channels is limited by the insufficient deep semantic attributes inherent in RGB images, which constrains model performance. The complete MCM outperforms reconstructing spatial patches of CLIP features, demonstrating the effectiveness of our proposal.

**Masking strategy.** Table 5 shows that random masking outperforms block-wise masking. Random masking encourages the model to integrate global unmasked channels to predict the masked ones, enhancing semantic coordination modeling across channels. In contrast, block-wise masking leads the model to overly rely on local unmasked blocks, making it difficult to capture long-range dependencies.

**Encode channel e.** The results in Table 6 indicate that the participation of the encode channels in both forward and backward propagation benefits MCM training. As training progresses, the features on the encode channels are dynamically adjusted to better collaborate with other channels, helping the model gain a deeper understanding of the complex contextual and semantic information between channels.

**Reconstruction range.** In Table 7, the performance of reconstructing all channels is superior to that of only reconstructing the masked portions. MCM, by collaboratively reconstructing the information of all channels, encourages the model to learn high-quality global semantics from CLIP.

**Scalability.** To further evaluate the effectiveness and scalability of MCM, we conduct experiments using the smaller ViT-S/16 [5] as the backbone. Since existing methods lack directly comparable results based on ViT-S, we reproduce the classic MAE method with both the raw pixel and CLIP features as reconstruction targets for comparison. In addition, MCM with pixel reconstruction is included in the comparison (Table 8). The results in Table 9 demonstrate that MCM achieves the best performance on all three benchmarks, showcasing its strong scalability. MCM’s focus on semantic continuity consistently extends its advantages to models of varying scales. Due to GPU memory constraints, we do not experiment with larger models such as ViT-L or ViT-H.

**Figure 3:**
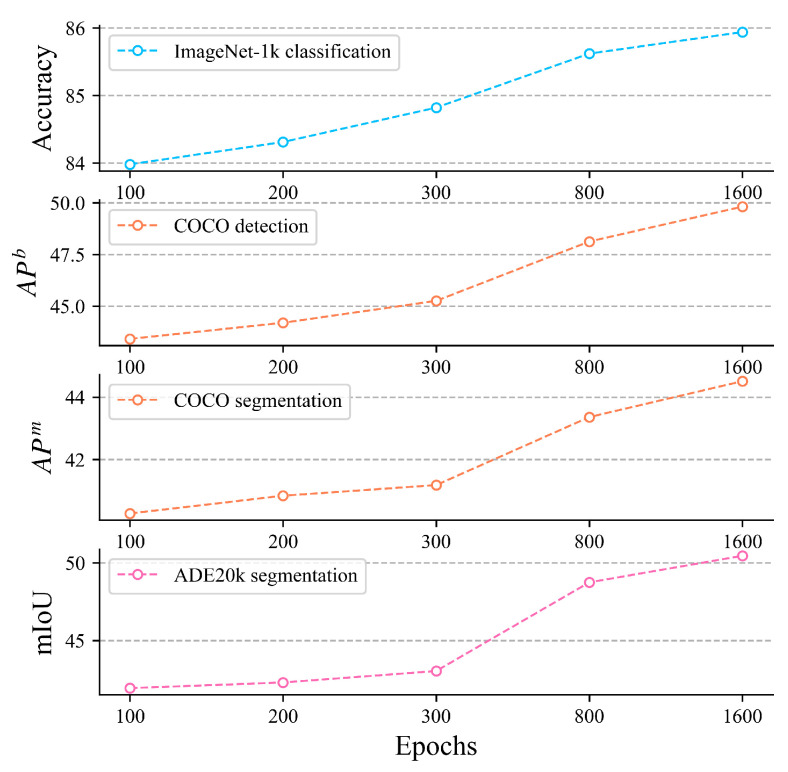
Performance of MCM pre-trained for different epochs on various downstream tasks.

**Pre-training epochs.** Figure 3 illustrates the fine-tuning results of MCM pre-trained for different epochs on various downstream tasks, demonstrating that adequately trained MCM effectively captures CLIP’s advanced semantics. As the number of pre-training epochs increases, MCM’s performance improves consistently across all tasks. Within the first 800 epochs, the model shows significant performance gains; however, the improvement becomes more moderate afterward. Interestingly, the growth in segmentation tasks outpaces that in other tasks. This could be attributed to MCM’s ability to learn advanced semantics, which is particularly beneficial for segmentation tasks, enabling it to better capture intricate object boundaries and global semantic relationships.

### 4.4. Visualization

We randomly select a subset of ImageNet-1k validation images and visualize the attention maps using the DINO visualization technique [27]. Due to the complexity of existing CLIP feature reconstruction methods and their reliance on additional learning modules [16,32,33], it is challenging to directly evaluate the impact of CLIP features on representation learning. Therefore, we compare a simplified reproduction of the MCM paradigm reconstructing raw pixels and the MIM paradigm reconstructing CLIP high-level features, both based on the MAE framework.

**Figure 4:**
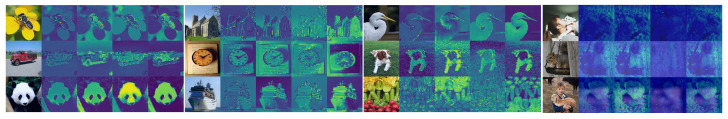
Attention maps of the last self-attention layer for ViT-B models pre-trained with different methods for 300 epochs. For each set, from left to right, the images correspond to the following: the original image, MAE with pixel reconstruction along the spatial patch, MAE with CLIP feature reconstruction along the spatial patch, MCM with pixel reconstruction along the channel semantics, and MCM with CLIP feature reconstruction along the channel semantics. To clearly showcase the attention distribution, all images are resized to 960×960 pixels.

As shown in Figure 4, MAE reconstructing CLIP features demonstrates more focused attention on the object compared to MAE but still struggles to completely eliminate background interference. Moreover, due to the limited semantic expression of pixels in the channel dimension, MCM reconstructing raw pixels exhibits more scattered attention, covering the entire scene. In contrast, the proposed MCM method significantly concentrates attention on the primary objects, validating that encouraging the model to learn semantic continuity across channels effectively enhances its understanding of CLIP high-level semantics and exhibits superior discriminative ability.

### 4.5. Discussion and Insights

The superior performance of our MCM can be attributed to two crucial design choices: (1) the explicit channel-wise masking that encourages semantic continuity modeling, and (2) the semantic-rich CLIP-derived reconstruction targets, guiding the model towards meaningful semantic representation rather than low-level pixel recovery. Ablation experiments clearly support this reasoning, showing substantial performance drops when either of these key components is replaced or removed.

## 5. Conclusions

This paper proposes a novel yet simple Masked Channel Modeling (MCM) pre-training paradigm for visual representation learning. Unlike reconstructing raw pixels or features in spatial patches, MCM leverages the contextual semantics of unmasked channels to reconstruct masked channels. This forces the model to focus on semantic continuity across channels, enabling it to learn more discriminative representations. MCM randomly masks most channels in each patch and replaces the masked part with independent encode channels and mask channels during encoding and decoding, respectively. Considering the limited semantic information in raw pixel-based channels, we use CLIP advanced features as targets to guide the model in learning higher-quality representations. Extensive experiments demonstrate the effectiveness and superiority of MCM. In the future, we will explore different learning components, helping MCM’s representation learning to improve with shorter training times. We also highlight that our approach is not limited to vision tasks. The MCM framework provides a broader methodology for learning semantic dependencies across feature dimensions, which may be applicable in interdisciplinary settings such as hyperspectral analysis, multi-sensor fusion, and biomedical imaging.

## Figures and Tables

**Table 1:** Performance comparison of different methods on various downstream tasks. Considering the differences in fine-tuning settings across various methods, we reproduce fine-tuning on COCO and ADE20K under the same experimental standards using the official pre-trained weights. Due to GPU memory limitations, we only fine-tune base-level models. The cells highlighted in pink correspond to our proposed method and the bold numbers indicate the best results in each column.

Methods	Venue	Backbone	Target	Epoch	ImageNet-1k	COCO		ADE20k
					**Top-1 Acc.**	${\mathrm{AP}}^{b}$	${\mathrm{AP}}^{m}$	**mIoU**
Contrastive Learning								
MoCo v3 [26]	ICCV’21	ViT-B [5]	Momentum	600	83.2	43.7	39.1	44.7
DINO [27]	ICCV’21	ViT-B	Momentum	300	82.8	-	-	44.6
Masked Image Modeling								
BEiT [2]	arXiv’21	ViT-B	DALLE [38]	800	83.2	46.8	41.7	44.6
MAE [3]	CVPR’22	ViT-B	Pixel	300	82.7	41.2	37.6	41.2
MAE [3]	CVPR’22	ViT-B	Pixel	1600	83.6	47.3	42.4	47.0
SimMIM [28]	CVPR’22	ViT-B	Pixel	800	83.8	47.4	41.8	47.8
MaskFeat [8]	CVPR’22	ViT-B	HOG	1600	84.0	47.6	42.3	47.3
LocalMIM [29]	CVPR’23	ViT-B	HOG	1600	84.0	47.7	42.2	47.1
SemMAE [30]	NeurIPS’22	ViT-B	Attention	800	83.4	45.6	40.9	44.9
PixMIM [31]	arXiv’23	ViT-B	Frequency	800	83.5	47.8	42.6	47.3
MVP [32]	ECCV’22	ViT-B	CLIP	300	84.4	48.4	42.9	48.3
MILAN [33]	arXiv’22	ViT-B	CLIP	400	85.4	49.5	43.3	48.8
MR-MAE [16]	IJCV’24	CViT-B [36]	CLIP	400	85.8	**49.8**	44.3	49.6
Masked Image Modeling + Contrastive Learning								
iBOT [34]	arXiv’21	ViT-B	dVAE [39] + Momentum	1600	84.0	48.1	41.8	47.9
BootMAE [35]	ECCV’22	ViT-B	Feature + Pixel	800	84.2	47.3	42.3	47.3
Masked Channel Modeling								
MCM	-	ViT-B	CLIP	300	84.8	45.3	41.2	43.0
MCM	-	ViT-B	CLIP	1600	**85.9**	**49.8**	**44.5**	**50.5**

**Table 2:** MCM ablation experiments. We report fine-tuning accuracy on ImageNet-1k, $AP^{b}$ and $AP^{m}$ on COCO, and mIoU on ADE20K. MCM is pre-trained for 300 epochs. The default settings are marked in pink. The bold numbers indicate the best results in each column. Different masking ratios γ. Same as tables below.

γ	Acc.	${\mathrm{AP}}^{b}$	${\mathrm{AP}}^{m}$	mIoU
60%	83.94	44.42	40.62	42.87
75%	84.82	**45.26**	**41.18**	43.03
80%	**84.87**	45.07	40.73	**43.14**
90%	84.19	44.66	40.32	42.66

**Table 3:** Numbers of ViT blocks in decoder.

Nums.	Acc.	${\mathrm{AP}}^{b}$	${\mathrm{AP}}^{m}$	mIoU
1	84.69	45.15	40.86	42.97
2	**84.82**	**45.26**	**41.18**	**43.03**
4	84.68	45.07	40.88	42.83
8	84.45	44.73	40.28	42.18

**Table 4:** Different reconstruction targets.

Target	Acc.	${\mathrm{AP}}^{b}$	${\mathrm{AP}}^{m}$	mIoU
pixel via patch	82.74	41.22	37.58	41.24
pixel via channel	81.61	39.87	36.29	40.06
CLIP via patch	84.31	44.84	40.67	42.93
CLIP via channel	**84.82**	**45.26**	**41.18**	**43.03**

**Table 5:** Different masking strategies.

Strategy	Acc.	${\mathrm{AP}}^{b}$	${\mathrm{AP}}^{m}$	mIoU
random	**84.82**	**45.26**	**41.18**	**43.03**
block-wise	84.41	44.94	40.86	42.75

**Table 6:** Encode channel configurations.

Config.	Acc.	${\mathrm{AP}}^{b}$	${\mathrm{AP}}^{m}$	mIoU
w/o forward	84.47	44.89	40.76	42.65
w/forward	**84.82**	**45.26**	**41.18**	**43.03**

**Table 7:** Reconstructed channel ranges.

Channel	Acc.	${\mathrm{AP}}^{b}$	${\mathrm{AP}}^{m}$	mIoU
masked	83.66	43.34	39.16	41.85
all	**84.82**	**45.26**	**41.18**	**43.03**

**Table 8:** Comparison with state-of-the-art Masked Channel Modeling methods on downstream tasks.

Method	Backbone	ImageNet-1k Acc. (%)	COCO AP (bbox)	ADE20K mIoU (%)
MAE [3]	ViT-B	83.4	48.4	46.2
Liu et al. [14]	ViT-B	85.6	49.2	49.8
ChA-MAEViT [15]	ViT-B	85.4	49.1	49.5
MCM (ours)	ViT-B	**85.9**	**49.8**	**50.5**

**Table 9:** Downstream results of various methods using ViT-S as the backbone and pre-trained for 300 epochs.

Methods	Target	IN1k	COCO		ADE20k
		**Acc.**	${\mathrm{AP}}^{b}$	${\mathrm{AP}}^{m}$	**mIoU**
MAE	Pixel	77.82	36.42	32.48	36.20
MAE	CLIP	79.26	40.03	35.94	39.15
MCM	Pixel	77.42	36.87	32.92	36.15
MCM	CLIP	**79.56**	**40.35**	**36.16**	**39.24**

## Data Availability

Data are contained within the article.

## Appendix A. Full Experimental Setups

Unless otherwise stated, all experiments are implemented on the PyTorch 2.1.0 platform and conducted on eight 24 GB RTX 3090 GPUs (NVIDIA Corporation, Santa Clara, CA, USA).

**Pre-training.** The training set of ImageNet-1k [17] is used for representation learning. Each image is resized to 224×224 pixels. MCM employs a plain ViT-B/16 [5] as the encoder and a decoder consisting of two layers of 768-dimensional ViT blocks. The random masking ratio γ is set to 75%. Except for the batchsize of 1024, the other pre-training configurations are in Table A1, following [3]. The fixed 2D sine–cosine positional embeddings are added to both the encoder and decoder inputs. All ViT blocks are initialized by xavier_uniform [40]. We use the batchsize of 1024 and the linear *lr* scaling rule [41]: lr=baselr×batchsize/256.

**Table A1:** Pre-training settings on ImageNet-1k.

Configs.	Pre-Training
optimizer	AdamW [42]
base learning rate	$1.5\times 10^{-4}$
weight decay	0.05
optimizer momentum	$\beta_{1},\beta_{2}=0.9,0.95$ [43]
learning rate schedule	cosine decay [44]
warmup epochs [41]	40
augmentation	RandomResizedCrop

**Fine-tuning on ImageNet-1k [17].** For image classification, we report the Top-1 accuracy on the ImageNet-1k validation set with fine-tuning protocol following previous practices [2,3]. Fine-tuning is performed with a batchsize of 1024 for 100 epochs. The detailed configurations are, respectively, shown in Table A2, which also follow MAE [3].

**Table A2:** Fine-tuning settings on ImageNet-1k.

Configs	Fine-Tuning
optimizer	AdamW
base learning rate	$1\times 10^{-3}$
weight decay	0.05
optimizer momentum	$\beta_{1},\beta_{2}=0.9,0.999$
layer-wise lr decay [2]	0.75
learning rate schedule	cosine decay
warmup epochs	5
augmentation	RandAug (9, 0.5) [45]
label smoothing [46]	0.1
mixup [47]	0.8
cutmix [48]	1.0
drop path [49]	0.1

**Fine-tuning on COCO [18].** Experiments for detection and segmentation are conducted on COCO [18]. We take Mask R-CNN [19] with FPN [20] as the detector with 1024 × 1024 resolution, following common practices [3]. We end-to-end fine-tune for 1× schedule (12 epochs) with the batchsize of 8. The other hyper-parameters follow the default settings of the detector. The full fine-tuning details are shown in Table A3. We adopt a multi-step scheduler to adjust the learning rate, which is reduced by 10× at the 8-th and 11-th epoch. We report box AP ($AP^{b}$) for object detection and mask AP ($AP^{m}$) for instance segmentation. The above experiments are implemented by the detectron2 [21] and the ViTDet [22] codebases.

**Table A3:** End-to-end fine-tuning settings on COCO.

Configs	COCO
optimizer	AdamW
base learning rate	$4\times 10^{-5}$
weight decay	0.1
optimizer momentum	$\beta_{1},\beta_{2}=0.9,0.999$
batchsize	8
layer-wise lr decay	0.75
learning rate schedule	multi-step scheduler
training epochs	12
drop path	0.1

**Fine-tuning on ADE20k [23].** The semantic segmentation experiments are conducted using the UperNet [24] framework for end-to-end supervised fine-tuning on ADE20K [23] with 512 × 512 resolution. The UperNet is trained for 160k iterations with a batchsize of 16, while other hyper-parameters remain at the default settings. The full implementation details is in Table A4. We turn on the relative position bias and initialize them with zero. The mean Intersection over Union (mIoU) on the ADE20K validation set is reported. These experiments are implemented using the mmsegmentation [25] library.

**Table A4:** End-to-end fine-tuning settings on ADE20k.

Configs	ADE20k
optimizer	AdamW
base learning rate	$1\times 10^{-4}$
weight decay	0.05
optimizer momentum	$\beta_{1},\beta_{2}=0.9,0.999$
batchsize	16
layer-wise lr decay	0.75
learning rate schedule	cosine decay
warmup iters	1500
training iters	160,000
drop path	0.1
